# Self-Esteem and Adolescent Bullying/Cyberbullying and Victimization/Cybervictimization Behaviours: A Person-Oriented Approach

**DOI:** 10.5964/ejop.5379

**Published:** 2022-08-31

**Authors:** Anna L. Palermiti, Maria G. Bartolo, Pasquale Musso, Rocco Servidio, Angela Costabile

**Affiliations:** 1 Department of Cultures, Education and Society, University of Calabria, Rende, Italy; 2 Department of Educational Sciences, Psychology, Communication, University of Studies of Bari “Aldo Moro”, Bari, Italy; Bangor University, Bangor, United Kingdom

**Keywords:** self-esteem, bullying, cyberbullying, victimization, cybervictimization, adolescents, person-oriented approach

## Abstract

Although previous studies seemed to recognize negative associations between self-esteem and bullying/cyberbullying and victimization/cybervictimization behaviours, the findings are controversial. The current study tried to shed light on this issue by using a person-oriented approach among Italian adolescents. Participants included 936 students aged 13-16 years. Different domains of self-esteem and bullying/cyberbullying and victimization/cybervictimization behaviour during the previous 2-3 months were assessed through a self-administered questionnaire. The results suggested four self-esteem profiles, i.e., school/family-oriented, consistently high, self-derogation, and body/peer-oriented. Students in the consistently high self-esteem profile seemed to be more protected against bullying/cyberbullying and victimization/cybervictimization behaviours compared to those in the self-derogation profile. The findings showed that among adolescents there is a degree of heterogeneity in the self-esteem domain associated with different levels of bullying/cyberbullying and victimization/cybervictimization behaviour. This suggests that different domains of self-esteem and their interdependencies play a crucial role during adolescence, with consequences also in terms of diverse patterns of active and passive aggressive behaviour.

Bullying and cyberbullying are two social phenomena that involve many children and youth. Both refer to repeatedly intentional, systematic, and aggressive behaviours manifested by an individual or a group of peers against a victim in a context of power imbalance (Smith, 2014). However, bullying concerns a vis-a-vis relationship, while cyberbullying pertains to the use of digital devices.

Bullying occurs in two different forms: direct (e.g., physical attacks or verbal offences) and/or indirect (e.g., social exclusion or the spreading of malicious rumours). It revolves around persons who play a specific role in a group, such as bully, victim, reinforcer of the bully, assistant of the bully, defender of the victim, and outsider (Salmivalli, Lagerspetz, Björkqvist, Österman, & Kaukiainen, 1996). Cyberbullying maintains some of the main characteristics of traditional bullying such as the intentionality and the imbalance of power, but it occurs in cyberspace or digital environments (e.g., social networks, chats, blogs), thus swiftly reaching a far wider audience. Like bullying, cyberbullying can manifest itself in different forms such as online harassment, denigration, happy slapping, trickery, and impersonation, and it is characterized by anonymity, stimulating more disinhibited behaviours and concealing cyberbullies’ identity from their victims (Kokkinos & Antoniadou, 2019; Lei et al., 2020).

Most researchers use the terms bullying and cyberbullying to denote both the active and passive sides of this aggressive/violent behaviour, but some prefer to adopt a clearer distinction by using *bullying/cyberbullying* to identify the active side of the phenomenon and *victimization/cybervictimization* to identify the passive one (e.g., Chen & Wei, 2011; Rodríguez-Enríquez, Bennasar-Veny, Leiva, Garaigordobil, & Yañez, 2019). This conceptual distinction is also adopted in the present study.

A growing number of studies on aggressive behaviours have investigated risk and protective factors related to bullying/cyberbullying and victimization/cybervictimization and have identified self-esteem as one of the most central of these factors (see, for example, Beltrán-Catalán, Zych, Ortega-Ruiz, & Llorent, 2018; Palermiti, Servidio, Bartolo, & Costabile, 2017; Tsaousis, 2016). Self-esteem is the product of a lifelong developmental process (see Mroczek & Little, 2014). It determines the overall assessment of a person’s value and can be considered an essential component of well-being. From this perspective, self-esteem may acquire a fundamental motivational function that can either activate or inhibit certain aspects of a person’s developmental trajectories (Harter & Whitesell, 2003), with high levels of self-esteem operating as protective factors and low levels increasing vulnerability to peer aggression and mental health problems (Ybrandt & Armelius, 2010).

However, the nature of the relation between self-esteem, on the one hand, and bullying/cyberbullying and victimization/cybervictimization, on the other, is still controversial (Lei et al., 2020, for a meta-analysis). In most cases, findings have highlighted a negative relation between self-esteem and victimization experiences, with high levels of self-esteem serving as protective factors (for the latest meta-analysis, see Tsaousis, 2016). Other studies have shown that both victims (Kowalski & Limber, 2013) and bullies (e.g., Jankauskiene, Kardelis, Sukys, & Kardeliene, 2008) have low levels of self-esteem. Other studies still have found bullies to have high levels of self-esteem (e.g., Salmivalli, Kaukiainen, Kaistaniemi, & Lagerspetz, 1999) or have found no association between self-esteem and bullying (Olweus, 1993; Pearce &Thompson, 1998). Research focusing on cyberbullying and cybervictimization has yielded similar controversial results (Cénat et al., 2014; Kowalski & Limber, 2013; Palermiti et al., 2017; Patchin & Hinduja, 2010).

Different explanations have been adduced to account for these results. Two of the main explanatory hypotheses are the *low self-esteem hypothesis* (Donnellan, Trzesniewski, Robins, Moffitt, & Caspi, 2005), suggesting that aggressive behaviours are an expression of youths’ low self-esteem, and the *disputed self-esteem hypothesis* (Baumeister, Bushman, & Campbell, 2000), arguing that children and youths adopt bullying behaviour because others threaten their self-esteem. However, these hypotheses do not account for the controversial nature of the results provided by the literature.

An alternative explanation is related to the approach that most studies have used to date. Most researchers have investigated the relation between self-esteem and bullying/cyberbullying and victimization/cybervictimization by using a variable-oriented approach with the self-esteem variable as the basic conceptual and analytic unit. As a consequence, they have considered the properties of the samples to be internally homogeneous. In practice, the variable-oriented approach, which analyses associations between variables based on aggregate data and average series across individuals, can misrepresent both the heterogeneity of the groups and individual patterns of development (von Eye & Bergman, 2003; von Eye & Bogat, 2006) and it is likely that it does not adequately reflect reality (Morin, Bujacz, & Gagné, 2018). Thus, the variable-centred approach can provide important information on the average differences between people, but it is often inaccurate and ignores variations in individuals’ experiences (Moses & Williford, 2017). On the other hand, a person-oriented approach (Bergman & Trost, 2006) has the advantage of allowing individuals to be classified into meaningful distinct groups based on their response patterns, whereby individuals within a group will be more similar than individuals across different groups (Moreira, Inman, Cloninger, & Cloninger, 2021). This makes it possible to address the multifaceted, varied nature of individual experiences. Despite these advantages, few studies so far have used a person-oriented approach to investigate how bullying/cyberbullying and victimization/cybervictimization behaviours are related to different self-esteem profile groups. As mentioned, this perspective may explain the current controversial findings in the field and require more investigation.

One reason for the underuse of the person-oriented approach may be the extensive use of global measures of self-esteem according to an exclusive low ↔ high unidimensional model. However, the literature shows how it is possible to conceptualize self-esteem not as a global concept but as a multidimensional construct including different domains. DuBois, Felner, Brand and George (1999) stated that self-evaluations find correspondence with the major settings or contexts of life, such as family, school, peer relations, physical appearance, and sports/athletics. This conceptualization was also supported by other studies (e.g., Brighi, Guarini, Melotti, Galli, & Genta, 2012; Harter, 2012) that similarly considered different domains of self-esteem, such as the educational, socio-relational, physical, and athletic. All these domains are particularly important during adolescent development. Indeed, adolescence is one of the most sensitive periods of life in terms of self-esteem processes, owing to the rapid physical, psychological, and social changes that are normally involved in this developmental phase. New relationships with family, school, and peers, and new issues related to peer acceptance and physical appearance make adolescents more susceptible to experiences and views that can largely influence their self-image (see Twenge & Campbell, 2001) and, consequently, their personal and social assets (e.g., skills and competencies). In view of this, since bullying/cyberbullying and victimization/cybervictimization phenomena may originate from diverse ecological conditions in different contexts (Bartolo, Palermiti, Servidio, Musso, & Costabile, 2019), it may be critical to investigate how different domains of self-esteem (e.g., family, school, peer relations) can be specifically linked with diverse forms of active and passive aggressive behaviour.

Starting from such premises, this study utilized a person-oriented approach to examine how bullying/cyberbullying and victimization/cybervictimization levels differed across different self-esteem profiles. To obtain such profiles we analysed similarities and differences among adolescent students with respect to the connections between self-esteem domain-related variables (DuBois et al, 1999). This enabled us, for example, to assign certain individuals a positive self-image in one domain but a negative self-image in another, without necessarily damaging their overall self-esteem level (von Soest, Wichstrøm, & Kvalem, 2016; Wichstrøm, & von Soest, 2016), thereby improving our understanding of adolescent aggressive behaviour development.

## Method

As already mentioned, the present study aimed to examine how self-esteem is associated with bullying/cyberbullying and victimization/cybervictimization behaviours in Italian adolescent students. To better account for the heterogeneity of individual developments, we adopted a person-oriented perspective (Bergman & Trost, 2006; Moreira et al., 2021) that focused on the identification of self-esteem profiles based on scores obtained by using a multi-domain (peer relations, school, family, physical appearance, sports/athletics) self-esteem measure (DuBois et al., 1999). Then, we assessed how self-esteem profiles were associated with bullying/cyberbullying and victimization/cybervictimization behaviours. We were generally guided by the hypothesis that self-esteem profiles characterized by high levels of self-esteem in diverse domains may mitigate involvement in bullying/cyberbullying and victimization/cybervictimization behaviours.

In exploring this hypothesis, gender and age were taken into account. These demographic variables have been shown to be differently associated with both bullying/cyberbullying and victimization/cybervictimization behaviours. In terms of gender differences, several studies have shown that males are usually more involved in bullying than females, especially when considering physical forms of bullying as opposed to the relational or verbal ones (Besag, 2006; Crick & Grotpeter, 1995). No major differences have been found with reference to victimization. Regarding cyberbullying/cybervictimization, studies have shown controversial findings: some studies have reported no differences (Hinduja & Patchin, 2007; Williams & Guerra, 2007), while others have found that males are more involved as cyberbullies (Li, 2006) and females as cybervictims (Guo, 2016; Calvete, Orue, Estévez, Villardón, & Padilla, 2010). In terms of age differences, a recent study (Tsaousis, 2016) reported that bullying tends to increase during childhood and early adolescence and to decline slightly only during the late adolescent years, while victimization decreases in the passage from childhood to adolescence. Cyberbullying and cybervictimization seem to follow different developmental patterns, with an increase over the years of secondary school (Smith & Slonje, 2010), probably due to easier access to digital communication tools and the Internet among older students.

### Participants

Participants included 980 ninth- and tenth-grade students recruited from 5 state high schools in southern Italy. Only a small and acceptable proportion of them (*n* = 44; 4.5%) had missing information pertaining to one or more of the study variables. These participants were excluded from the analyses. Our final sample thus consisted of 936 adolescents (males = 48.6% and females = 51.4%) aged 13 to 16 years (*M* = 14.49, *SD* = 0.50). The majority of participants were Italian (93.0%), had cohabiting parents (87.8%), and came from middle-class backgrounds (76.4%) – less than 2% of the adolescents’ parents had an elementary school education and less than 22% had a university or postgraduate education.

### Procedure

The whole procedure was performed in accordance with the Italian Psychological Association’s ethical principles (http://www.aipass.org/node/11560). Participants’ parents were informed – via a letter from the principal of each school – about the purpose of the research, the voluntary nature of participation, and the anonymity of the responses. Parents provided written informed consent to their son’s or daughter’s participation. Furthermore, adolescent participants were required to submit a signed assent form in order to take part in the study. Participants had about 30 mins to complete an anonymous online survey during class time and could withdraw at any time.

### Measures

#### Socio-Demographics

Participants were asked to indicate their gender, age, ethnicity, and socio-economic status (SES), as well as their parents’ marital status and education.

#### Self-Esteem Questionnaire

The 31-item self-report Italian version of the Self-Esteem Questionnaire (SEQ; DuBois et al., 1999; Melotti & Passini, 2002) was used to assess different domains of self-esteem: peer relations (seven items; e.g., “I am as good as I want to be at making new friends”), school (seven items; e.g., “I feel OK about how good of a student I am”), family (eight items; e.g., “I get along with my family as well as I would like to”), physical appearance (four items; e.g., “I like my body just the way it is”), sports/athletics (five items; e.g., “I am as good at sports/physical activities as I want to be”). Items were scored on a Likert-type scale ranging from 1 (*totally disagree*) to 4 (*totally agree*). For each subscale, a mean score was computed. Cronbach’s alpha values for peer relations (α = .88), school (α = .87), family (α = .77), physical appearance (α = .87), and sports/athletics (α = .87) were consistent with prior research.

#### Florence Bullying/Victimization Scales

The Florence Bullying and Victimization Scales (FBVSs; Palladino, Nocentini & Menesini, 2016) were used to assess traditional bullying and victimization behaviours during the previous 2–3 months. Each scale contains 10 items (e.g., “I called someone names” for bullying and “I have been called names” for victimization). Both scales consist of three subscales: physical behaviours, verbal behaviours, and indirect-relational behaviours. Previous research supported the use of the FBVSs as second order measures to obtain global scores for bullying and victimization. Items were scored on a Likert-type scale ranging from 1 (*never*) to 5 (*several times a week*). For each scale, a mean score was computed. Cronbach’s alpha values for physical (α = .82), verbal (α = .77), indirect-relational (α = .68), and global bullying (α = .85) scores as well as physical (α = .62), verbal (α = .69), indirect-relational (α = .65) and global victimization (α = .78) scores were consistent with prior research.

#### Florence CyberBullying/CyberVictimization Scales

The Florence CyberBullying/CyberVictimization Scales (FCBVSs; Palladino et al., 2016) were used to assess cyberbullying and cybervictimization behaviours during the previous 2–3 months. Each scale contains 14 items (e.g., “I have stolen personal data, such as images and photos, in order to reuse them” for cyberbullying and “Personal data, such as images and photos, have been stolen from me in order to reuse them” for cybervictimization). Both scales consist of four subscales: written-verbal, visual, impersonation, and exclusion. Previous research also supported the use of the FCBVSs as second order measures to obtain global scores for cyberbullying and cybervictimization. Items were scored on a Likert-type scale ranging from 1 (*never*) to 5 (*several times a week*). For each scale, a mean score was computed. Cronbach’s alpha values for written-verbal (α = .88), visual (α = .86), impersonation (α = .91), exclusion (α = .83), and global cyberbullying (α = .95) scores as well as written-verbal (α = .79), visual (α = .72), impersonation (α = .80), exclusion (α = .75), and global cybervictimization (α = .90) scores were consistent with prior research.

### Data Analysis

We followed three main steps to carry out the data analysis. First, we computed descriptive statistics for the key study variables including means and standard deviations, skewness and kurtosis indices, the minimum and maximum values of standardized scores, and Pearson’s bivariate correlations. This allowed verification of the univariate normality of the distributions. Whenever required, non-normally distributed variables were transformed to improve normality and extreme outliers. Furthermore, the Mahalanobis distance and Mardia’s multivariate kurtosis coefficient were used to test multivariate normality and identify other potential multivariate outliers. Then, the final descriptive statistics for the study variables were computed.

Second, to identify self-esteem profiles of adolescent students, we conducted a cluster analysis based on the standardized scores of SEQ subscales (peer relations, school, family, physical appearance, and sports/athletics). Specifically, at an initial step, we carried out agglomerative hierarchical cluster analyses, using Ward’s method based on the squared Euclidean distance (Aldenderfer & Blashfield, 1984) and examining solutions from two to six clusters, to determine the most appropriate number of clusters. The criteria used to choose this number included the theoretical meaningfulness of each cluster, parsimony, and explanatory power. With regard to explanatory power, the cluster solution had to explain at least 26% of the variance in each of the SEQ dimensions (see Cohen, 1988). Then, the study participants were grouped by *K*-means cluster analysis procedures and the standardized mean values of the SEQ grouping variables describing the characteristics of each identified cluster were calculated. To check the validity and stability of the solution, a multivariate analysis of variance (MANOVA) on the five SEQ dimensions (the dependent variables) by the cluster groups (the independent variable) was performed and the replicability of the solution was tested. As indicated by Breckenridge (2000), data were randomly divided into two subsets (A and B) and cluster analyses were newly conducted for each of them. Then, subset B was classified into clusters according to the cluster centres derived from subset A and the agreement between the two subset B solutions was computed using Cohen’s kappa, with higher agreement indicative of a more stable cluster solution.

Third, to compare the bullying/cyberbullying and victimization/cybervictimization variables in relation to the self-esteem profile groups, we carried out a MANOVA with self-esteem profiles, gender, and age (dummy coded: 0 = 13–14 years; 1 = 15–16 years) as independent variables and bullying/cyberbullying and victimization/cybervictimization behaviours as dependent variables.

## Results

### Descriptive Statistics

We initially computed descriptive statistics. As is commonly the case in the investigated field of study (Palladino et al., 2016), bullying/cyberbullying and victimization/cybervictimization variables were strongly non-normal (see Tabachnick & Fidell, 2013), with skewness values ranging from 3.49 to 8.99, kurtosis values ranging from 19.98 to 98.53, and the maximum values of standardized scores ranging from 9.46 to 14.26. For these reasons, a transformation was applied for all these variables using the two-step approach to transform the non-normally distributed variables suggested by Templeton (2011), as the best choice. After re-calculating descriptive statistics for the transformed variables, the new distributions showed acceptable values (see Table 1). Furthermore, the multivariate inspection of the data revealed twenty-six (2.8%) potential multivariate outliers. However, after performing analyses with or without these cases, we found no substantial effect on the pattern of results. Thus, we retained these cases in the final sample.

**Table 1:** Means, Standard Deviations, Skewness, Kurtosis, Minimum/Maximum Values

Variable	1	2	3	4	5	6	7	8	9
*Self-esteem (domains)*									
1. Peer relations	—	.28***	.41***	.41***	.47***	.00	-.33***	-.01	-.16***
2. School		—	.37***	.24***	.21***	-.14***	-.13***	-.13***	-.14***
3. Family			—	.37***	.32***	-.19***	-.26***	-.19***	-.30***
4. Physical appearance				—	.53***	-.01	-.21***	-.07*	-.17***
5. Sports/athletics					—	-.01	-.19***	-.02	-.10**
6. Bullying						—	.41***	.50***	.33***
7. Victimization							—	.23***	.47***
8. Cyberbullying								—	.48***
9. Cybervictimization									—
Transformation	No	No	No	No	No	Yes	Yes	Yes	Yes
*M*	3.00	2.68	3.33	2.74	2.77	1.24	1.24	1.10	1.12
*SD*	0.50	0.63	0.54	0.79	0.67	0.35	0.32	0.22	0.22
Skewness	-0.03	-0.02	-0.20	-0.03	-0.06	0.73	0.68	1.80	1.18
Kurtosis	-0.19	-0.21	-0.42	-0.56	-0.30	-0.24	-0.41	2.18	0.45
Minimum standardized score	-3.12	-2.71	-3.19	-2.00	-2.65	-0.95	-0.96	-0.50	-0.69
Maximum standardized score	2.30	2.28	1.62	1.85	2.04	3.69	3.48	4.50	4.02

*Note.* Values are of standardized scores and bivariate correlations for the key study variables in their original or final transformed version (*n* = 936).

*p < .05. **p < .01. ***p < .001.

### Cluster Analysis

#### Identification of the Optimal Number of Clusters

Based on the initial agglomerative hierarchical cluster analyses and the a priori criteria, a four-cluster solution was found to be the most acceptable. On the one hand, the solution with two or three clusters explained less than 26% of the variability in at least one of the SEQ specific dimensions. On the other hand, solutions with five or six clusters violated the principle of parsimony, because they included clusters that represented slight variations compared to the four most interpretable clusters and did not have a clear theoretical meaning.

#### K-Means Cluster Analysis and Description of Self-Esteem Profiles

**Figure 1:**
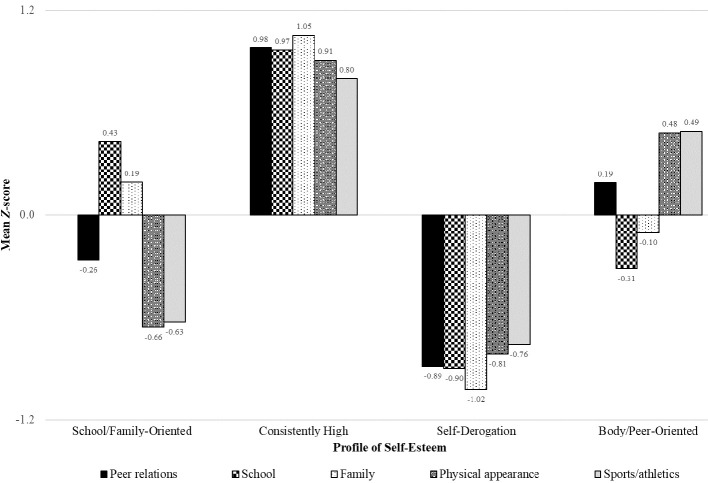
Mean Z-Scores *Note.* For the self-esteem domains of peer relations, school, family, physical appearance, and sports/athletics across the four self-esteem profiles.

After establishing the most appropriate number of clusters, the participants were clustered into four groups by *K*-means cluster analysis. Figure 1 shows the self-esteem profiles obtained. The first cluster (*n* = 223, 23.83% of the sample) was composed of students who scored moderately high on the self-esteem domains of school and family, moderately low on the self-esteem domain of peer relations, and low on the self-esteem domains of physical appearance and sports/athletics. The second cluster (*n* = 199, 21.26% of the sample) consisted of students scoring high on all self-esteem domains of peer relations, school, family, physical appearance, and sports/athletics. The third cluster (*n* = 217, 23.18% of the sample) comprised primarily students with low scores on all five self-esteem domains. The fourth cluster (*n* = 297, 31.73% of the sample) consisted of students scoring moderately high on the self-esteem domains of physical appearance, sports/athletics, and peer relations, and moderately low on the self-esteem domain of family and school. Thus, we found, in sequence, clusters representing school/family-oriented, consistently high, self-derogation, and body/peer-oriented self-esteem profiles (DuBois et al., 1999).

#### Validity and Stability of the Cluster Solution

The MANOVA computed on the five self-esteem domain variables revealed a significant multivariate effect of the cluster solution, Wilks’ Lambda = .12, *F*(15, 2562) = 197.19, *p* < .001, η^2^ = .51, indicating that about 51% of the variability was accounted for by group differences among the four clusters. Also, follow-up ANOVAs indicated that the four-cluster solution explained good percentages of variance for each variable related to the diverse self-esteem domains (41% of variability in peer relations, 46% in school, 49% in family, 50% in physical appearance, and 44% in sports/athletics). The replicability procedure indicated that the four-cluster solution was the best for both random subsets, A and B, and that the agreement between the two solutions of subset B was .73, indicating a substantial level of reliability.

### MANOVA on Bullying/Cyberbullying and Victimization/Cybervictimization Variables

#### Multivariate Test Statistic

The MANOVA on the bullying/cyberbullying and victimization/cybervictimization variables resulted in a significant multivariate effect of the self-esteem profiles, Wilks’ Lambda = .90, *F*(12, 2424) = 8.48, *p* < .001, η^2^ = .04, and gender, Wilks’ Lambda = .96, *F*(4, 916) = 10.54, *p* < .001, η^2^ = .04. None of the other main effects and two- or three-way interactions were statistically significant.

#### Follow-Up Univariate Analyses

Follow-up univariate analyses indicated that (a) levels of bullying/cyberbullying and victimization/cybervictimization behaviour differed significantly across self-esteem profiles, and (b) bullying/cyberbullying (but not victimization/cybervictimization) differed significantly across gender. Specifically, group comparisons based on Tukey’s Honestly Significant Difference (HSD) post hoc analyses for the four self-esteem profiles (see Table 2) and on Bonferroni adjustments for gender (see Table 3) revealed that:

**Table 2:** Univariate Analyses of Variance and Post Hoc Group

	MANOVA-Adjusted Means by Self-Esteem Profile					
Variable	School/Family-Oriented *n* = 223	Consistently High *n* = 199	Self-Derogation *n* = 217	Body/Peer-Oriented *n* = 297	*F*(3, 920)	η^2^
Bullying	1.19_a_	1.20_a_	1.31_b_	1.25_ab_	7.36***	0.02
Victimization	1.28_a_	1.12_b_	1.38_c_	1.20_d_	27.87***	0.08
Cyberbullying	1.08_a_	1.06_a_	1.15_b_	1.10_a_	8.35***	0.03
Cybervictimization	1.12_a_	1.04_b_	1.19_c_	1.11_a_	13.70***	0.04

*Note.* Comparisons based on Tukey’s tests for the four self-esteem profiles on bullying/cyberbullying and victimization/cybervictimization behaviours. Different subscripts (a, b, c, or d) within a row indicate significant differences (*p* < .05) between profile group means.

***p < .001.

**Table 3:** Univariate Analyses of Variance and Pairwise

	MANOVA-Adjusted Means by Gender			
Variable	Male *n* = 455	Female *n* = 481	*F*(1, 920)	η^2^
Bullying	1.31_a_	1.17_b_	32.54***	0.03
Victimization	1.25	1.24	0.00	0.00
Cyberbullying	1.11_a_	1.08_b_	4.03*	0.01
Cybervictimization	1.11	1.12	0.63	0.00

*Note.* Comparisons based on Bonferroni’s adjustments for gender on bullying/cyberbullying and victimization/cybervictimization behaviours. Different subscripts (a and b) within a row indicate significant differences (*p* < .05) between profile group means.

*p < .05. ***p < .001.

- Students in the self-derogation profile scored significantly higher on all bullying/cyberbullying (mean scores 1.31 and 1.15, respectively) and victimization/cybervictimization (1.38 and 1.19) behaviours than those in the other profiles (≤ 1.20 for bullying, ≤ 1.10 for cyberbullying, ≤ 1.28 for victimization, and ≤ 1.12 for cybervictimization), except for the comparison with the students in the body/peer-oriented profile in relation to bullying (1.31 vs. 1.25);
- Students in the consistently high self-esteem profile scored significantly lower on victimization/cybervictimization behaviours (1.12 and 1.04) than those in the other profiles (≥ 1.20 for victimization, and ≥ 1.11 for cybervictimization), while they scored similarly on bullying/cyberbullying behaviours compared to those in the school/family-oriented (1.20 vs. 1.19 for bullying and 1.06 vs. 1.08 for cyberbullying) and body/peer-oriented profiles (1.20 vs. 1.15 for bullying and 1.06 vs. 1.10 for cyberbullying);
- Students in the body/peer-oriented profile scored significantly lower on victimization than those in the school/family-oriented profile (1.20 vs. 1.28), while both groups scored similarly on bullying/cyberbullying (1.25 vs. 1.19 for bullying and 1.10 vs. 1.08 for cyberbullying) and cybervictimization (1.11 vs. 1.12) behaviours;
- Male students scored significantly higher on bullying/cyberbullying behaviours than female students (1.31 vs. 1.17 for bullying and 1.11 vs. 1.08 for cyberbullying), while both groups scored similarly on victimization/cybervictimization behaviours (1.25 vs. 1.24 for victimization and 1.11 vs. 1.12 for cybervictimization).

## Discussion

The main purpose of the current study was to examine the association of self-esteem profiles with bullying/cyberbullying and victimization/cybervictimization behaviours in adolescence. Differently from most previous studies, the added value was to apply a person-oriented approach to characterize our participants based on different domains of self-esteem (DuBois et al., 1999). This enabled us to better consider their heterogeneity, by identifying different subgroups of participants with specific configurations of self-esteem. Results indicated that four different self-esteem profiles (i.e., school/family-oriented, consistently high, self-derogation, and body/peer-oriented) could be extracted and highlighted substantial differences between these profiles in terms of behavioural levels for bullying/cyberbullying and victimization/cybervictimization.

Findings on self-esteem profiles were largely consistent with those of previous studies (e.g., DuBois et al., 1999; Salmivalli et al., 1999), reporting both consistently high and self-derogation self-esteem profiles. The school/family-oriented and the body/peer-oriented profiles were theoretically meaningful and interpretable. From a developmental-ecological perspective, school, family, body-image, and peer relations are highly significant domains in adolescents’ lives (e.g., Ricciardelli & McCabe, 2001). Moreover, these domains may be considered interdependent across settings (DuBois et al., 1999), with specific experiences and views in one domain (e.g., family or peer relations) tending to have corresponding impacts on those associated with related domains (e.g., school or body-image). Furthermore, such experiences and views may be more prevalent in one area of life (e.g., relations with adults in the family and at school) than another depending on the personal and environmental context in which adolescents are developing.

With regard to the associations between self-esteem profiles and bullying/cyberbullying and victimization/cybervictimization behaviours, the results showed that students classified in the self-derogation profile seemed to be more at risk of being involved in both bullying/cyberbullying and victimization/cybervictimization behaviours compared to those in the other profiles. This may complement findings in recent previous studies showing that lower levels of self-esteem are associated with higher risks of bullying and victimization (Tsaousis, 2016). One exception is the non-significant difference between students in the self-derogation and body/peer-oriented profiles with respect to bullying. The emphasis on body- and peer-oriented self-evaluation domains may significantly increase students’ concerns about their concrete adaptive social experiences (DuBois et al., 1999), thereby increasing the risk of involvement in problematic behaviours, like bullying.

Students in the consistently high self-esteem profile seemed to be more protected against bullying/cyberbullying and victimization/cybervictimization behaviours compared to those in the self-derogation profile. This seems like the other side of the coin with respect to what was already mentioned above. Also, adolescents in the consistently high self-esteem profile seemed to be more protected against victimization/cybervictimization behaviours compared to those in the school/family- and body/peer-oriented profiles, while there were no significant differences in terms of bullying/cyberbullying behaviours. The synergistic interactions between the self-esteem domains in the consistently high profile (DuBois et al., 1999) may have the potential of considerably enriching personal and social skills and strategies that safeguard against situations of victimization. However, this could be only partially true and associated with specific skills and strategies with regard to school/family- or body/peer-oriented profiles. Thus, it appears that, in terms of reducing bullying/cyberbullying behaviours, it is important to establish uniform patterns of self-evaluation in at least one specific area (i.e., school and family or body-image and peer relations), if not all of them, as this would help prevent the expression of greater levels of aggression and antisocial behaviour as in the self-derogation profile.

Generally, students in the school/family-oriented profile presented the same levels of bullying/cyberbullying and cybervictimization behaviour as students in the body/peer-oriented profile, but the latter adolescents seemed to be more protected against victimization behaviours than the former. The emphasis on body- and peer-oriented domains of self-evaluation may increase—as already specified above—the risk of involvement in bullying behaviours but, at the same time, it may also facilitate the development of strategies to reduce victimization behaviours and events.

The findings were also examined in the light of demographic variables such as gender and age as well as their interactions with the self-esteem profiles. Only gender showed any significant direct influence. Specifically, boys reported higher levels of bullying/cyberbullying than girls. This is consistent with several studies (e.g., Li, 2006; Olweus, 1993). However, no differences emerged with regard to victimization/cybervictimization. This result is also in line with previous studies (e.g., Williams & Guerra, 2007), although other researchers found that girls are more involved in victimization/cybervictimization behaviours than boys (Smith et al., 2008). The results illustrating that levels of bullying/cyberbullying and victimization/cybervictimization behaviour were similar in our two age subgroups of adolescents seem to support studies that explain how bullying/cyberbullying and victimization/cybervictimization behaviours tends to change during childhood, but stabilize during early and middle adolescence (Tsaousis, 2016).

Taken together, these findings are important because they provide new evidence for an issue that previous literature has demonstrated to be either inconclusive or controversial. Although some previous studies agreed that there is a potential negative association between self-esteem and peer bullying/cyberbullying and victimization/cybervictimization behaviours, most of them were based on a variable-oriented approach. The results from the current study, however, show that among adolescents there is a self-esteem-domain heterogeneity associated with different levels of bullying/cyberbullying and victimization/cybervictimization behaviours. This suggests that different domains of self-esteem and their interdependencies play a crucial role during adolescence. Indeed, during that period the increased importance of peer relations and physical appearance may make adolescents more inclined to focus on their self-image (Twenge & Campbell, 2001), with consequences also in terms of diverse patterns of aggressive behaviour.

The weaknesses of the present study should also be taken into account. First, we were not able to analyse our data according to a multilevel approach owing to the completely anonymous online administration of the questionnaire. Future research should be conducted to examine this issue. Second, we did not analyse the data in terms of the different forms of bullying/cyberbullying and victimization/cybervictimization owing to space limitations. It would be important to understand how diverse self-esteem profiles are associated with specific forms of active-passive aggressive behaviour. Finally, we only considered gender and age as variables related to bullying/cyberbullying and victimization/cybervictimization behaviours in addition to self-esteem profiles. Nevertheless, the literature suggests the need to consider other factors as well (Bartolo et al., 2019; Cook, Williams, & Guerra, 2010).

Despite these limitations, our study presents important implications for current policies and practices, suggesting guidelines for designing educational programs aimed at preventing bullying/cyberbullying and victimization/cybervictimization phenomena. Generally, anti-bullying agencies should develop more effective individual-centred prevention programs (Tsaousis, 2016). With regard to victimization/cybervictimization, it would be important to design intervention programs that help adolescents to build their own self-confidence in all important domains of their life. This implies interventions at different levels: individual, family, and school (Tenuta, Bartolo, Diano, & Costabile, 2020). With regard to bullying/cyberbullying, such intervention programs should guarantee the improvement of adolescent’s self-images in at least one of the relevant areas of life, with slightly greater attention to the areas of the family and school. As a conclusive recommendation, in the case of more targeted interventions in a specific sphere, it is advisable, and even simpler, to directly involve significant people who fit within that living space, such as parents, teachers or peers (for example, in the role of mentors).
